# Supplementary material for: The Systems Biology Research Tool: evolvable open-source software
Source: BMC Syst Biol. 2008 Jun 29;2:55. doi: 10.1186/1752-0509-2-55 (PMC2446383; doi:10.1186/1752-0509-2-55)
Supplement: Additional file 1 — SBRT Archive. An archive of the current version of the Systems Biology Research Tool. [file 1752-0509-2-55-S1.zip › sbrt-1.4.0/doc/developers_guide/api/sbrt/kernel/fba/proc/FluxIntervalProcess.html]

FluxIntervalProcess


|  |  |  |
| --- | --- | --- |
| **Overview** \| **Package** \| **Class** \| **Use** \| **Tree** \| **Deprecated** \| **Index** \| **Help** | |  |
| **PREV CLASS**   **NEXT CLASS** | **FRAMES**    **NO FRAMES**     **All Classes** |  |
| SUMMARY: NESTED \| FIELD \| CONSTR \| METHOD | DETAIL: FIELD \| CONSTR \| METHOD |  |


---


## sbrt.kernel.fba.proc Class FluxIntervalProcess

```
java.lang.Object
  sbrt.kernel.fba.proc.FbaProcess<java.util.Map<java.lang.String,Interval>>
      sbrt.kernel.fba.proc.FluxIntervalProcess
```

**All Implemented Interfaces:**: KernelProcess<java.util.Map<java.lang.String,Interval>>, MonitoredProcess<java.util.Map<java.lang.String,Interval>>

---

``` public final class FluxIntervalProcess extends FbaProcess<java.util.Map<java.lang.String,Interval>> implements MonitoredProcess<java.util.Map<java.lang.String,Interval>> ```

This class is used to perform flux interval experiments.

**Author:**
:   This class was written and documented by Jeremiah Wright
    while in the Wagner lab.

---

**Constructor Summary**

`FluxIntervalProcess(FluxIntervalComputer computer)`
  
          Constructs a new flux range experiment.


| **Method Summary** | |
| --- | --- |
| `Fluxome` | `getFluxome()`             Returns the fluxome used in this experiment. |
| `ProgressMonitor` | `getProgress()`             Returns a progress monitor for this experiment. |
| `void` | `run(ResultsManager<java.util.Map<java.lang.String,Interval>> resultsManager)`             Computes the flux ranges of the reactions with the current names and adds them to the provided results manager. |
| `void` | `setReactionNames(java.util.Set<java.lang.String> reactionNames)`             Sets the names of reactions for which flux ranges are to be computed. |

**Methods inherited from class sbrt.kernel.fba.proc.FbaProcess**

`toString`

**Methods inherited from class java.lang.Object**

`clone, equals, finalize, getClass, hashCode, notify, notifyAll, wait, wait, wait`

**Constructor Detail**

### FluxIntervalProcess

```
public FluxIntervalProcess(FluxIntervalComputer computer)
```

:   Constructs a new flux range experiment.

    **Parameters:**: `computer` - the computer with which all flux ranges will be computed.


**Method Detail**

### setReactionNames

```
public void setReactionNames(java.util.Set<java.lang.String> reactionNames)
```

:   Sets the names of reactions for which flux ranges are to be computed.

    :   **Parameters:**: `reactionNames` - the names of reactions for which flux ranges are to be computed.

---


### getFluxome

```
public Fluxome getFluxome()
```

:   Returns the fluxome used in this experiment.

    :   **Specified by:**: `getFluxome` in class `FbaProcess<java.util.Map<java.lang.String,Interval>>`
    :   **Returns:**: the fluxome used in this experiment.

---


### getProgress

```
public ProgressMonitor getProgress()
```

:   Returns a progress monitor for this experiment.

    :   **Specified by:**: `getProgress` in interface `MonitoredProcess<java.util.Map<java.lang.String,Interval>>`
    :   **Returns:**: a progress monitor for this experiment.

---


### run

```
public void run(ResultsManager<java.util.Map<java.lang.String,Interval>> resultsManager)
```

:   Computes the flux ranges of the reactions with the current names and
    adds them to the provided results manager.

    :   **Specified by:**: `run` in interface `KernelProcess<java.util.Map<java.lang.String,Interval>>`
    :   **Parameters:**: `resultsManager` - the results manager to which the computed flux ranges will be added.


---


|  |  |  |
| --- | --- | --- |
| **Overview** \| **Package** \| **Class** \| **Use** \| **Tree** \| **Deprecated** \| **Index** \| **Help** | |  |
| **PREV CLASS**   **NEXT CLASS** | **FRAMES**    **NO FRAMES**     **All Classes** |  |
| SUMMARY: NESTED \| FIELD \| CONSTR \| METHOD | DETAIL: FIELD \| CONSTR \| METHOD |  |


---
